# I-124 codrituzumab imaging and biodistribution in patients with hepatocellular carcinoma

**DOI:** 10.1186/s13550-018-0374-8

**Published:** 2018-03-05

**Authors:** Jorge A. Carrasquillo, Joseph A. O’Donoghue, Volkan Beylergil, Shutian Ruan, Neeta Pandit-Taskar, Steven M. Larson, Peter M. Smith-Jones, Serge K. Lyashchenko, Norihisa Ohishi, Toshihiko Ohtomo, Ghassan K. Abou-Alfa

**Affiliations:** 1 Department of Radiology, Memorial Sloan Kettering Cancer Center, 1275 York Avenue, New York, NY 10065 USA; 2 Department of Medical Physics, Memorial Sloan Kettering Cancer Center, 1275 York Avenue, New York, NY 10065 USA; 3 Department of Psychiatry and Behavioral Science, Stony Brook University Hospital, 101 Nicolls Road, Stony Brook, NY 11794 USA; 4 Department of Radiology, Stony Brook University Hospital, 101 Nicolls Road, Stony Brook, NY 11794 USA; 5 Chugai Pharmaceutical Co., Ltd., 1-1 Nihonbashi-Muromachi 2-Chome Chuo-ku, Tokyo, 103-8324 Japan; 6 Department of Medicine, Memorial Sloan Kettering Cancer Center, 1275 York Avenue, New York, NY 10065 USA

**Keywords:** Codrituzumab, I-124, Antibody, Glypican, Hepatocellular

## Abstract

**Background:**

I-124 codrituzumab (aka GC33), an antibody directed at Glypican 3, was evaluated in patients with hepatocellular carcinoma (HCC). Fourteen patients with HCC underwent baseline imaging with I-124 codrituzumab (~ 185 MBq, 10 mg). Seven of these patients undergoing sorafenib/immunotherapy with 2.5 or 5 mg/kg of cold codrituzumab had repeat imaging, with co-infusion of I-124 codrituzumab, as part of their immunotherapy treatment. Three patients who progressed while on sorafenib/immunotherapy were re-imaged after a 4-week washout period to assess for the presence of antigen. Serial positron emission tomography (PET) imaging and pharmacokinetics were performed following I-124 codrituzumab. An ELISA assay was used to determine “cold” codrituzumab serum pharmacokinetics and compare it to that of I-124 codrituzumab. Correlation of imaging results was performed with IHC. Short-term safety assessment was also evaluated.

**Results:**

Thirteen patients had tumor localization on baseline I-124 codrituzumab; heterogeneity in tumor uptake was noted. In three patients undergoing repeat imaging while on immunotherapy/sorafenib, evidence of decreased I-124 codrituzumab uptake was noted. All three patients who underwent imaging after progression while on immunotherapy continued to have I-124 codrituzumab tumor uptake. Pharmacokinetics of I-124 codrituzumab was similar to that of other intact IgG. No significant adverse events were observed related to the I-124 codrituzumab.

**Conclusions:**

I-124 codrituzumab detected tumor localization in most patients with HCC. Pharmacokinetics was similar to that of other intact iodinated humanized IgG. No visible cross-reactivity with normal organs was observed.

**Electronic supplementary material:**

The online version of this article (10.1186/s13550-018-0374-8) contains supplementary material, which is available to authorized users.

## Background

Therapeutic applications of antibody-based pharmaceuticals continue to expand [1]. The development of antibodies based on direct cytotoxicity, effect on checkpoint blockade, or delivery of drugs as antibody drug conjugates (ADC) continues to advance. Given the large number of candidate antibodies for these applications, it would be useful to develop tools and strategies for prioritization or early termination of which reagents should be moved forward in phase 2 or 3 trials.

There has long been an interest in using radiolabeled antibodies for tumor targeting—initially, to develop these as imaging reagents—but more recently, there has been an interest in using radiolabeled antibodies in a theranostic setting to inform drug development [2–4]. Initial radiolabeled antibody studies focused on the use of radionuclides that were single-photon emitters such as I-131- and In-111-labeled reagents. These single-photon-emitting isotopes had limitations in terms of lack of quantitation and also in terms of sensitivity and resolution of the scanners. These limitations are largely overcome by the use of positron-emitting radiopharmaceuticals that allow quantitation, and have higher sensitivity for detection and improved resolution. A limited number of studies have shown the ability of I-124-labeled antibodies to detect and quantitate tumor localization [5–9]. Other positron emitters have been utilized for antibody labeling, including Ga-68, Cu-64, and Y-86, but these are limited by the short *T*_1/2_ in comparison to the pharmacokinetics of intact antibody [10, 11]. Zr-89, a long-lived positron emitter that has been used successfully both clinically [4, 12–16] and in pre-clinical testing subsequent to the start of our trial [17, 18], could have been a reasonable alternative for radiolabeling. Our choice of I-124 for labeling was justified based on two reasons: (1) concerns that using residualizing radiometals such as Zr-89 would result in higher liver background, as has been found with other radiometals (In-111 and Lu-177), thus lowering tumor-to-normal liver contrast, and (2) the slow internalization rate of GPC3 when targeted by a radiolabeled antibody [17, 18]. Furthermore, we did not have access to Zr-89 for clinical use from our cyclotron at that time. It is also worth noting that the mechanism of immunotherapy response with this antibody is based on antigen-dependent cytotoxicity (ADCC), which depends on the antibody being available on the cell surface.

Glypican-3 (GPC3) is a member of the glypican family, a group of heparan sulfate proteoglycans linked to the cell surface through a glycosylphosphatidylinositol anchor [19, 20]. Glypicans play an important role in cell growth, differentiation, and migration. GPC3 protein is expressed in a wide variety of tissues during development, but the expression in most adult tissues is suppressed. Recently, it was shown that GPC3 is highly expressed in the majority of hepatocellular carcinomas (HCCs) [21, 22]. Furthermore, GPC3 expression has been correlated with poor prognosis in HCC [23].

Codrituzumab is a recombinant, humanized monoclonal antibody (mAb) that binds to human GPC3 with high affinity (Kd of 0.673 nM) [24]. The nonclinical pharmacological assessments have shown that codrituzumab elicited antibody-dependent cellular cytotoxicity through human peripheral blood mononuclear cells (PBMCs) as well as mouse effector cells against GPC3-expressing human HCC and hepatoblastoma cell lines in vitro. They also showed anti-tumor activities in several mouse xenograft models inoculated with human HCC cell lines expressing GPC3 [25].

These pre-clinical findings have led to a phase I clinical trial in the USA and another in a Japanese population with advanced HCC. Both of these studies showed that codrituzumab was well tolerated with no dose limiting toxicity or maximal tolerated dose reached [22, 26]. In addition, preliminary evidence for potential anti-tumor activity was observed in patients with high expression of GPC3 [22].

This report is part of a phase I study using escalating doses of codrituzumab in conjunction with sorafenib in patients with advanced HCC (Clinical trials.gov identifier NCT00976170). The objectives for this report using I-124 codrituzumab were to explore the distribution profile, dosimetry, and detailed pharmacokinetics. Additionally, we aimed to determine if escalating amounts of cold codrituzumab blocked uptake of I-124 codrituzumab and evaluate whether patients who failed treatment with codrituzumab still expressed GPC3 using I-124 codrituzumab imaging. The immune therapeutic portions of this study have been previously reported, and limited portions of the imaging results and pharmacokinetics have been described previously [27]. This study focuses on the imaging aspects, biodistribution, and dosimetry.

## Methods

### Patient eligibility and protocol design

This was a prospective, single-center imaging study using I-124 codrituzumab antibody for the first time as part of a “Phase I, open-label, multi-center, dose escalation study of the safety, tolerability, and pharmacokinetics of codrituzumab in combination with sorafenib (Nexavar^®^) in patients with advanced or metastatic hepatocellular carcinoma (NCT00976170).” This study was reviewed and approved by Memorial Sloan Kettering (MSK)’s institutional review board (IRB), and all patients provided verbal and written informed consent. Partial data on imaging results and pharmacokinetics, as well as full toxicity and response data, have been reported previously as part of the phase I immunotherapy trial [27].

Seventeen patients signed informed consent, but three did not receive antibody due to progression prior to starting treatment, cholangiocarcinoma on re-review of biopsy, and decision to not participate. Fourteen consecutive patients received a total of 24 injections of I-124 codrituzumab; demographic data is shown in Table 1. The mean age was 65.1 years (range 50–82 years). Twelve patients were males (85.7%) and two were females (14.3%). Other inclusion criteria were as previously described [27].

**Table 1:** Patient demographics, pathology, and imaging

Patient no.	Age/sex	Race	Child-Pugh score	Etiology	TNM	AJCC path grading	I-124 Czb baseline 10 mg	I-124 Czb second injection (mg/kg)	I-124 Czb third injection 10 mg	Day between IHC sample and I-124 Czb second injection	IHC H membrane	IHC H cytoplasmic	SUVmax tumor of biopsied lesion
1	63/M	White	A	None	IIIB	Poorly differentiated	Yes	5	ND	41	4	7	7
2	71/M	White	A	None	IV	Cannot be assessed	Yes	ND	ND	18	155	84	30.9
4	67/M	White	B	None	IV	Cannot be assessed	Yes	ND	ND	34	145	15	7.3
5	69/F	Asian	A	None	IIIC	Well differentiated	Yes	5	ND	46	30	25	5.8
6	64/M	White	A	None	IIIB	Cannot be assessed	Yes	ND	ND	11	0	15	NA
7	82/M	White	A	None	IIIB	Moderately differentiated	Yes	5	Yes	46	40	13	6.7
8	62/M	White	A	HepC	IV	Cannot be assessed	Yes	ND	ND	21	4	6	6.8
9	81/M	Asian	A	HepB	N/A	Well differentiated	Yes	2.5	Yes	38	5	85	8.1
10	58 M	Asian	A	HepB	I	Undifferentiated	Yes	ND	ND	10	165	150	12.8
11	68/F	A/A	A	HepC	IV	Cannot be assessed	Yes	ND	ND	14	1	25	4.9
13	58/M	A/A	A	HepC, Alcoholic	IIIA	Poorly differentiated	Yes	ND	ND	ND	ND	ND	ND
14	60/M	White	A	HepC	IV	Cannot be assessed	Yes	2.5	Yes	27	2	62	8.4
16	59/M	White	A	Alcoholic	IV	Moderately differentiated	Yes	2.5	ND	ND	ND	ND	ND
17	50/M	White	A	HepB	IV	Well differentiated	Yes	2.5	ND	ND	ND	ND	ND

*Abbreviations*: *path* pathology, *Czb* codrituzumab, *A/A* African-American, *TNM* tumor, node, metastasis staging system, *AJCC* American Joint Committee on Cancer, *IHC* immunohistochemistry, *ND* not done

All patients received a baseline injection of I-124 codrituzumab followed by serial imaging and biodistribution. Following completion of imaging, patients started treatment with weekly intravenous immunotherapy with codrituzumab (2.5 or 5 mg/kg) and oral sorafenib twice daily. In total, 7 of the 14 patients underwent a second injection of I-124 codrituzumab co-infused with their fourth weekly immunotherapy injection of codrituzumab. The I-124 codrituzumab was co-infused during the last 10–15 min of the non-labeled codrituzumab immunotherapy injection, with both injections terminating at the same time. Three patients received a third injection of I-124 codrituzumab 4–6 weeks after stopping codrituzumab and sorafenib for documented progression, in order to assess if antigen continued to be present. Because the repeat scans were optional, some patients opted not to have a second or third scan.

Within 2–24 h prior to injection of I-124 codrituzumab and for 14 additional days, patients received five drops of saturated potassium iodide solution (SSKI) to block a thyroid uptake. Following I-124 codrituzumab baseline and second injections, all patients underwent similar blood draws for pharmacokinetics. In addition, they underwent positron emission tomography/computed tomography (PET/CT) imaging on four occasions during the ensuing 7 days. Those undergoing a third imaging study had no pharmacokinetics or blood draws, and only two sets of images were obtained post-injection.

### Radiolabeling of codrituzumab

Codrituzumab is a recombinant, fully humanized mAb that binds to human GPC3. The codrituzumab was supplied by Chugai Pharmaceuticals Co., Ltd. (Tokyo, Japan) in sterile, single-use vials containing 10 mL solution at a concentration of 20 mg/mL of codrituzumab. This non-labeled antibody was used for the immunotherapy portion of the protocol and for iodination with I-124. The I-124 codrituzumab was manufactured in the cyclotron of MSK’s radiochemistry core in accordance with an investigational new drug (IND) application. I-124 was obtained from the cyclotron (*n* = 18) [28] or from IBA Molecular North America (Totowa, NJ, USA) (*n* = 6).

As per protocol, formulations containing a nominal 185 MBq of I-124 codrituzumab, 10 mg of codrituzumab, and 5 mL of 0.9% sodium chloride containing 5% human serum albumin were used. One exception was a low-yield reaction that contained 137 MBq of I-124 codrituzumab with good immunoreactivity. In brief, ~ 370 MBq of I-124 was incubated with 10 mg of codrituzumab in an iodogen tube. The mixture was then purified through an anion-exchange column followed by terminal sterilization through a 0.22-μm filter. The activity of the final product was assayed in a dose calibrator, and cold antibody was added to yield an activity containing ~ 185 MBq/10 mg. All products passed pyrogen and sterility testing. A total of 24 radiolabeled products were formulated for patient injections. The mean injected activity was 181 ± 14 MBq. The mean radiochemical purity was 99.6 ± 0.79%, determined by ITLC with 10% trichloroacetic acid. All products were sterile and had a median of < 1.6 endotoxin units per milliliter with a final volume of 4.8 ± 0.3 mL. The median immunoreactivity as determined by a cell binding assay using HEP-G2 cells was 72.4% (range 60.5–86.4%), using a modification of the Lindmo method [29].

### Imaging

Patients were imaged using a Discovery DSTE PET/CT scanner in 2D mode. Images were reconstructed using conventional GE software and for standard processing of F-18 images; OSEM reconstruction was used. Patients underwent imaging within 1–4 h of infusion and 1, 2–3, and 4–6 days post-injection. Images from the mid-skull to proximal thighs were acquired for 5 min per field of view (FOV) on the day of injection and increased up to 8–10 min per FOV on the last day of imaging. The CT scans for co-registration and attenuation were performed at 140 KeV and 10 mA for all but one scan, which was acquired at 80 mA. All patients had their final images at a median of 5 days after tracer administration. Scans following the second infusion were obtained using similar parameters as the first injection. Following injection for the third scan (*n* = 3), image acquisition was obtained on the day of injection and at 2 to 3 days post-injection.

Images were read by an experienced nuclear medicine physician who was aware of the patient’s history and diagnostic CT scan findings (JAC). Localization in tumor was defined as focal accumulation greater than adjacent background in areas where physiologic activity is not expected. All images and maximum-intensity-projection (MIP) images were reviewed on a dedicated PET analysis workstation (HERMES Medical Solutions AB, Stockholm, Sweden). Volumes of interest (VOIs) were placed visually over structures of interest, and mean and maximum SUVs normalized to body weight [(nCi/mL activity in region)/(nCi-injected activity/body mass in grams)] were determined for the blood pool, liver, spleen, thyroid, small bowel, and large bowel. The % injected activity per gram (%IA/g) was derived from SUVs. VOIs over the liver, lung, spleen, and kidney were used to determine time-activity curves and residence times. Blood pharmacokinetic data was used to obtain blood residence time. Residence times were then entered into OLINDA/EXM using the standard models based on sex and best approximating body weight [30]. The delivered radiation dose in tissues and organs were then summarized.

### Pharmacokinetics

Pharmacokinetic analysis of I-124 codrituzumab was performed by drawing blood after injection within 10 min, 30 (± 5) min, 1 h (± 5 min), 2 h (± 30 min), 4 (± 1) h, 8 (± 1) h, and around the time of PET/CT at 1, 2–3, and 4–6 days after the end of I-124 codrituzumab. This pharmacokinetic analysis was performed for both the first and the second infusions. Samples were centrifuged, and the serum was aliquoted, weighed, and counted in a scintillation well counter (Wallac Wizard 1480 automatic gamma counter, PerkinElmer), with a standard of the injected activity and the decay-corrected %IA/L in serum calculated. The data was fit to a bi- or monoexponential function SAAM II application [31]. The fit parameters were used to determine concentration extrapolated to zero time (Co), the serum *T*_1/2_, volume of central compartment (*V*_c_), volume of distribution at steady state (Vdss), area underneath the curve (AUC), and clearance. The AUC was determined by trapezoidal integration up to the time of the last serum samples, and the terminal component was estimated using the fitted half-life. To determine the %IA in the plasma volume, the %IA/L at the end of infusion was multiplied by the patient’s estimated plasma volume determined from a nomogram [32]. To determine if the blood pool concentration derived from PET imaging correlated with counting of serum samples, a VOI was drawn over the atrial blood pool. The SUVmax in the blood was converted to %IA/L of the serum using the patient’s weight and hematocrit and compared to the concentration measured by direct blood sampling at the nearest time of imaging.

### ELISA for codrituzumab

We compared the differences in the clearance of the parent, non-labeled antibody versus the I-124 codrituzumab. The seven patients with a second I-124 codrituzumab injection that was co-infused with an immunotherapy dose of cold codrituzumab (2.5 or 5 mg/kg) had a serial serum sampling that was measured both in the gamma counters for determination of I-124 codrituzumab content and evaluated by ELISA to determine codrituzumab antibody concentration. In brief, a validated ELISA using human GPC3 core protein as a capture antigen and rabbit anti-codrituzumab antibody and a goat anti-rabbit IgG-HRP detector antibody was performed by Chugai Research Institute for Medical Science, Inc. (Kanagawa, Japan).

In order to compare the clearance rate from the blood of codrituzumab determined by the ELISA measurement (measuring predominantly non-radiolabeled codrituzumab) or based on the concentration of I-124 radioactivity (measuring I-124-labeled codrituzumab), the initial time post-injection measurement was taken as 100% and subsequent concentrations were normalized to the initial time. In addition, the data expressed as a percent of the injected activity per liter was previously presented [27]. In this paper, the AUC from the first time-point to the last measured time was integrated using trapezoidal integration and a paired *t* test was used to determine if any differences in AUC were noted between measurements based on radioactivity or ELISA.

### GPC3 expression

Immunohistochemistry (IHC) data was generated in the main “parent” sorafenib/immunotherapy protocol. Tumor expression of GPC3 was examined in biopsied specimens by IHC using mouse anti-human GPC3 mAb [33], generating an H score. IHC scoring was performed on biopsy samples obtained between 10 and 46 days (Table 1), and samples on three patients were not sufficient to be tested. IHC was performed and interpreted as previously described, assigning a score based on positive cell rate and staining intensity (cytoplasmic or cell membrane) (Additional file 1: Table S1) [22]. In 10 of 14 patients, lesions biopsied for IHC determination could be identified on PET/CT and underwent exploratory correlation with SUVmax.

In addition to IHC for GPC3 expression, soluble GPC3 fragments in circulation were also measured. Two anti-N-terminal fragment mAbs (designated GT30 and GT607) and two anti-C-terminal fragment mAbs (designated GT96 and M3C11) were used in combination in two different assays (sGPC3 GT30/GT607, sGPC3 M3C11/GT96) to detect full-length proteins or any possible cleavage fragments ([34], Pradier, Reis, Jukofsky, et al. Indian buffet process identifies 776 biomarkers of response to codrituzumab, submitted). In 7 out of 14 patients, soluble GPC3 values were measured prior to treatment and underwent exploratory correlation with SUVmax.

### Evaluation for toxicity

Patients were monitored for adverse events during the I-124 codrituzumab infusion and at each imaging time-point. Adverse events were graded using the National Cancer Institute toxicity criteria (V3.0).

### Statistics

Descriptive statistics including median or mean and standard deviation (mean ± SD) and Pearson correlation coefficient were performed. Comparison between groups was performed with either paired *t* test or ANOVA. All statistical analyses were performed with Sigma Stat 3.5 (Systat Software Inc., San Jose, CA, USA).

## Results

**Fig. 1:**
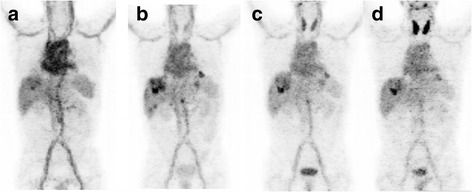
Patient 10 showed definite tumor accumulation in liver lesions at ~ 24 h and significant blood pool accumulation that decreased over time: 3 h (**a**), 22 h (**b**), 46 h (**c**), and 118 h (**d**). The highest SUVmax in the liver was 15.3 at 46 h. Normal organ distribution including the blood pool, liver, spleen, and kidney decreased over time. In contrast, free iodine accumulation in the thyroid increased over time

**Fig. 2:**
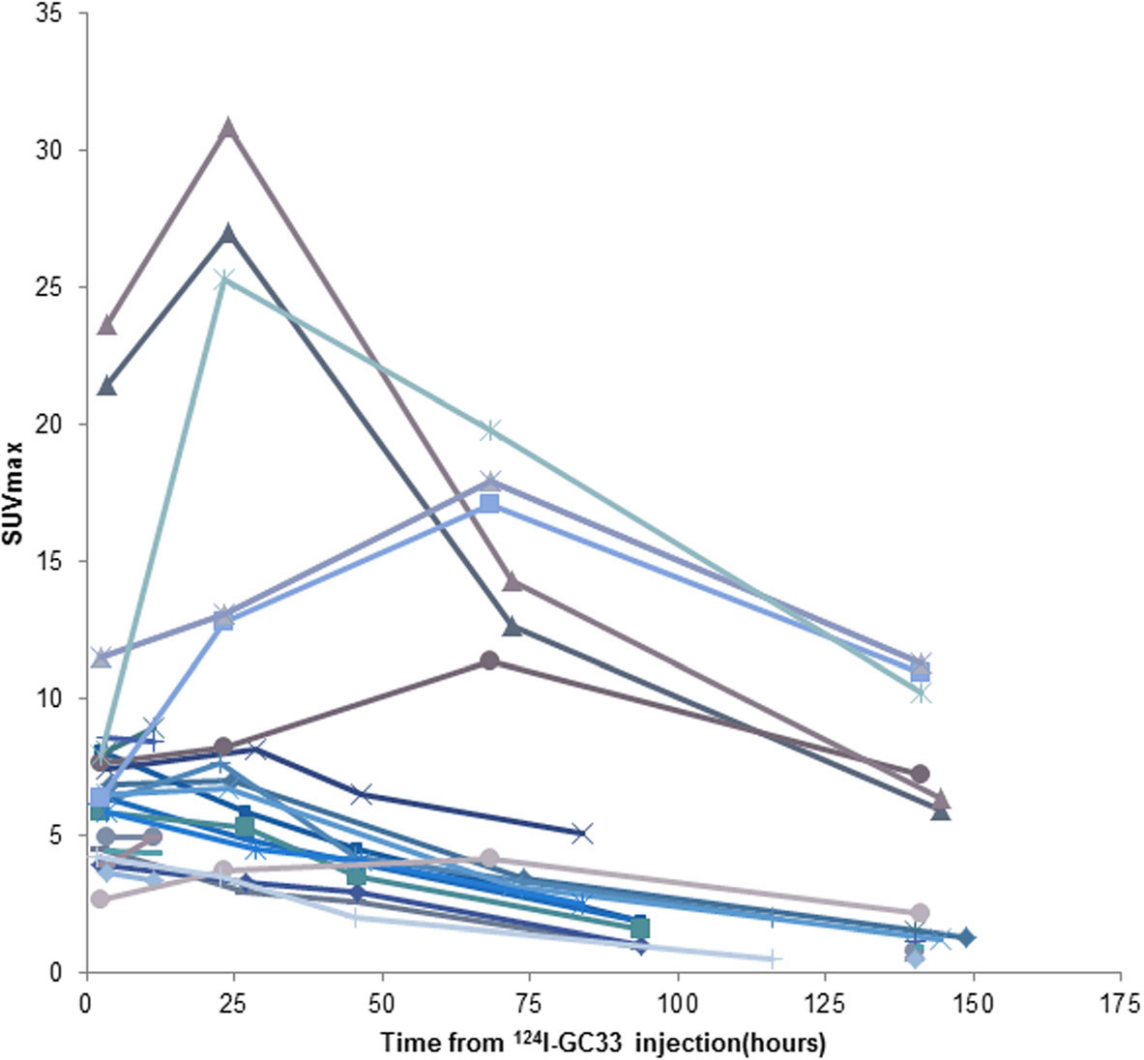
Each line represents a tumor in a single patient imaged over time; the tumor selected had the most intense uptake. Typically, maximal tumor uptake occurred within 24 h of tracer administration

**Fig. 3:**
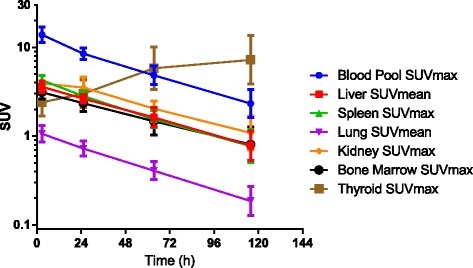
Summary data from the baseline study in all patients receiving I-124 codrituzumab. VOIs were performed over the various organs and tissues. The amount of uptake was expressed in SUVmax or mean as indicated in the figure legend. The curves show that with the exception of the thyroid, which accumulates free I-124 over time, all other organs had a gradual decrease that was very similar to the clearance rate from the blood

**Fig. 4:**
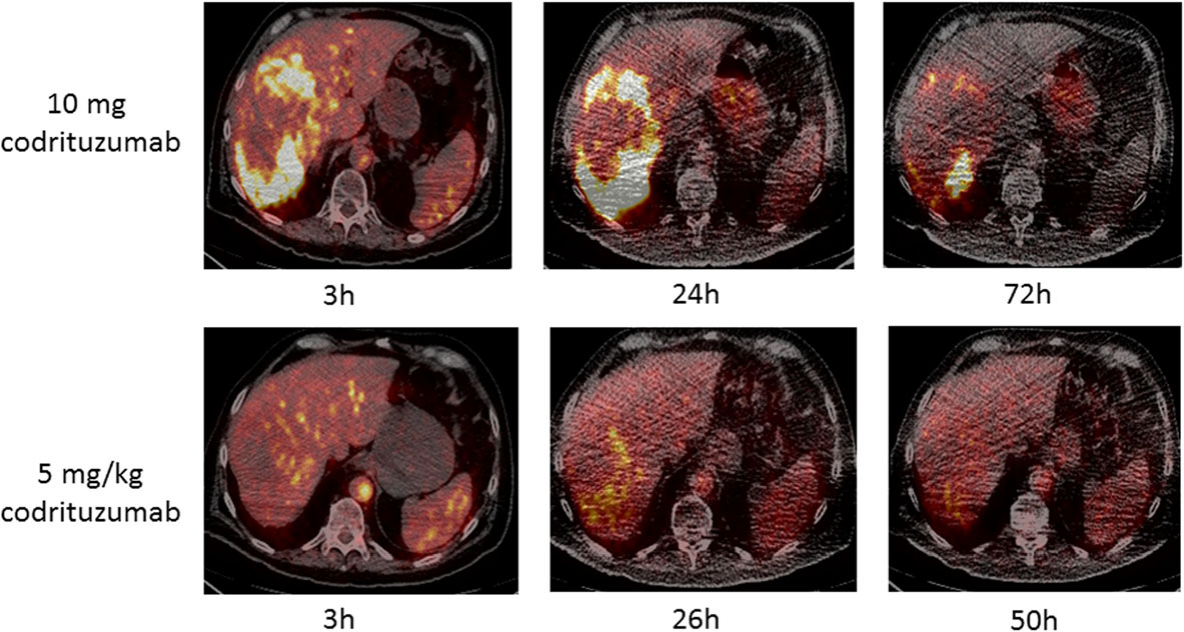
Patient 7, serial-fused I-124 codrituzumab-PET/CT baseline images following injection of 190 MBq of I-124 codrituzumab (10 mg of codrituzumab) (upper panel). Upper panel images show prominent heterogeneous uptake in a large right liver tumor lesion, with more uptakes in the periphery than those in the central region. The lower panel shows serial-fused I-124 codrituzumab-PET/CT images obtained after treatment with sorafenib and codrituzumab (5 mg/kg) for 4 weeks and after injection of 183 MBq I-124 codrituzumab (co-infused with 5 mg/kg of codrituzumab). All images are displayed at the same SUV and show marked decrease in intensity of uptake in the periphery. For example, at 24 to 26 h, the SUVmax decreased in the cold co-infused images (lower panel) from 20 to 40% of the 10 mg injected mass, and in the periphery, there was less of a drop in SUVmax to 80–90% of the 10 mg injected mass, probably because of central necrosis

A representative scan is shown in Fig. 1. Of the 14 patients who underwent a baseline scan, 13 had tumor-positive scan findings and 1 had no uptake above normal liver background as previously reported [27]. There were seven patients who had tumor-to-blood-pool ratios greater than 2 and an additional four that reached ratios greater than 1.0. In 6 of the 13 patients, the uptake was prominent (SUV > 9) (Fig. 1), and in 7, the uptake was just mildly above the background. Quantitative data from serial imaging showed that in most patients, tumor concentration peaked by 24 h and slowly decreased afterward (Fig. 2). Heterogeneity in tumor visualization was frequently observed with abnormalities noted on contrast CT with no corresponding uptake on PET imaging. There was no preferential normal organ accumulation with the exception of the thyroid (Fig. 1). Following injection, activity cleared from the blood and normal organs, generally in parallel with the decrease in the blood (Figs. 1 and 3). Uptake in the thyroid was the only organ that showed increase over time with the maximum uptake seen in patient 10 of 0.0172%IA/g. Uptake in the lung and spleen had no increase beyond the blood pool. Whereas in the normal liver, most of the uptake was accounted for by blood pool content as demonstrated by minimal increase in the organ-to-blood-pool ratio. In the kidney and marrow, a minor increase in ratios over time was observed, but while they were statistically significant, they were very small (Additional file 1: Figure S1). Repeat imaging following cold codrituzumab immunotherapy in seven patients showed marked quantitative decrease in the tumor uptake in two patients, and as described previously Fig. 4 [27], no blocking of uptake in normal tissue was observed.

### Pharmacokinetics

**Fig. 5:**
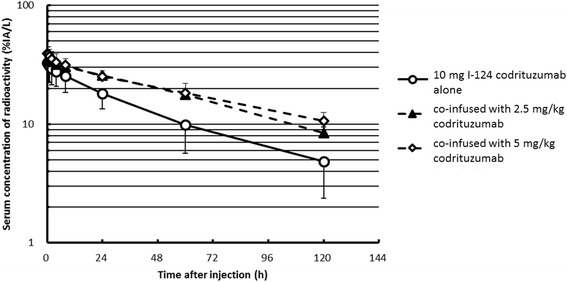
%IA/L of serum was plotted following injection of 10 mg I-124 codrituzumab alone (circle) or co-infused with 2.5 mg/kg (triangles) or 5 mg/kg (squares). The larger mass amount of antibody resulted in significant mass-dependent changes with longer half-life and greater AUC than the 10 mg injection (Table 2). There was an overlap of the clearance curves for the 2.5 and 5 mg/kg co-infused mass

The derived pharmacokinetics parameters are shown in Table 2. Serum clearance of I-124 codrituzumab is shown in Fig. 5. Serum concentration of radioactivity decreased biphasically in the majority of patients (*n* = 13 of 17). Following the first injection of I-124 codrituzumab (10 mg), the serum concentration decreased faster than with the second injection of I-124 codrituzumab co-infused with 2.5 and/or 5 mg/kg of cold codrituzumab.

**Table 2:** I-124 codrituzumab pharmacokinetics

Dose	Co observed %IA/L	Co estimated %IA/L	*V*_c_ (L)	*T*_1/2_ delayed (h)^a^	AUC based on fit (%IA × h/L)	Clearance (ml/h)	Vdss (L)
10 mg (*n* = 14)	32.9 ± 9.1	32.4 ± 9.2	3.399 ± 1.233	46.2 ± 12.58	1712 ± 438	61.8 ± 14.8	3.669 ± 1.281
2.5 mg/kg (total 130–240 mg) (*n* = 4)	35.3 ± 10.3	35.3 ± 10.5	3.033 ± 0.894	125.7 ± 129.3	3429 ± 1559	34.2 ± 15.4	2.924 ± 0.830
5 mg/kg (total 260–400 mg) (*n* = 3)	39.2 ± 5.24	37.3 ± 6.8	2.740 ± 0.464	99.7 ± 26.6	3956 ± 757	25.8 ± 4.5	3.504 ± 0.871

^a^In 14 studies, this represents the beta component of biexponential fit; in 7 studies, this represented the monoexponential fit. Fits were performed using SAAM software

The *V*_c_ derived from the serum measurements of patients receiving 10 mg of I-124 codrituzumab was not significantly different from the estimate based on the patients’ height and weight, measuring 3.399 L ± 1.233 vs. 3.045 ± 0.469, respectively (Wilcoxon signed-rank test *p* = 0.426). Paired comparison of *V*_c_ or Vdss for 10 mg and 2.5 or 5 mg/kg co-infusion were not significantly different (*p* = 0.351 and 0.744, respectively) (Table 2). When the measured serum concentration at initial time post-injection (Co) was multiplied by the estimated plasma volume, based on a nomogram, we found that the injected activity distributed in the serum accounted for 101.8 ± 2.8%.

The AUC in the serum for the combined group receiving 2.5 or 5 mg/kg of cold antibody was larger (3655 ± 1219 %IA × h/L) than that of their corresponding 10 mg I-124 codrituzumab baseline (1678 ± 501 %IA × h/L) (paired *t* test *p* = 0.004). The clearance was also higher in the baseline 10 mg infusion than when co-infused with 2.5 or 5 mg/kg of cold antibody (63.8 ± 17.4, 34.2 ± 15.4, and 25.8 ± 4.5 ml/h, respectively; *p* = 0.002, one-way repeated measure ANOVA). No difference in clearance was seen between the 2.5 and 5 mg/kg of antibody (Table 2).

No significant differences in clearance of radiolabeled antibody versus cold non-labeled antibody were noted, and time-activity curves expressed as %IA/L were compared and overlapped as previously described [27]. The AUC from initial to final serum measurement for either I-124 codrituzumab or ELISA-determined antibody concentration were not significantly different, with an AUC of 2362 ± 716 versus 2448 ± 1177 %IA × h/L, respectively (*p* = 0.753, paired *t* test).

The concentration in plasma determined by gamma counting of serum samples (gold standard) was compared to that estimated from PET images. The concentration of I-124 codrituzumab in the serum of all patients determined by VOI analysis of initial PET images was similar to that obtained from serum measurement on the gamma counter of overlapping time (28.6 ± 6.3 %IA/L vs. 28.8% ± 7.4 %IA/L; *n* = 14, *p* = 0.84 paired *t* test).

Soluble GPC3 values prior to treatment measured using two different combinations of anti-GPC3 mAbs were very small (20.51 pg/mL as minimal and 33.60 ng/mL as maximal values in seven evaluable patients) compared to the amount of injected antibody in the circulation. The degree of tumor uptake on SUVmax and soluble GPC3 values at baseline tend to correlate, especially soluble GPC3 values measured using anti-GPC3 C-terminal mAb pairs for codrituzumab, which showed higher correlation (Additional file 1: Figure S2).

IHC showed heterogenous antigen distribution in tumor specimens. The degree of tumor uptake based on SUVmax and IHC staining in either cytoplasm or membrane tended to correlate (Additional file 1: Figure S3).

### Correlation with clinical outcome

No correlation was observed between change in tumor volume and SUVmax (Additional file 1: Figure S4). No correlation was observed between time on study, time on study drug, or length of survival and degree of tumor uptake.

### Dosimetry results

Dosimetry estimates obtained from the initial study (*n* = 14) were averaged, as shown in Table 3. The major target organs were the thyroid (4.16 ± 2.19 cGy/37 MBq**)**, heart (3.85 ± 0.55 cGy/37 MBq), bladder wall (2.73 ± 0.27 cGy/37 MBq**)**, and liver (2.43 ± 0.38 cGy/37 MBq). In addition, the effective dose equivalent was 1.65 ± 0.21 cGy/37 MBq for the first study. Patients receiving a second study had greater dose deposited to most organs, with an effective dose equivalent that was slightly higher (2.13 ± 0.36 cGy/37 MBq).

**Table 3:** I-124 codrituzumab radiation dose estimates

	I-124 codrituzumab 10 mg (*n* = 14)		I-124 codrituzumab 2.5–5 mg/kg (*n* = 7)	
Target organ	Mean cGy/37 MBq	SD	Mean cGy/37 MBq	SD
Adrenals	1.14	0.17	1.57	0.33
Brain	0.60	0.14	0.84	0.19
Breasts	0.71	0.12	1.01	0.21
Gallbladder wall	1.18	0.18	1.60	0.33
LLI wall	0.88	0.19	1.21	0.26
Small intestine	0.90	0.19	1.25	0.27
Stomach wall	0.92	0.17	1.29	0.28
ULI wall	0.91	0.18	1.26	0.27
Heart wall	3.82	0.55	5.60	1.20
Kidneys	1.74	0.31	2.28	0.54
Liver	2.43	0.38	3.19	0.64
Lungs	1.87	0.34	2.44	0.48
Muscle	0.75	0.15	1.05	0.22
Ovaries	0.91	0.19	1.25	0.27
Pancreas	1.13	0.18	1.56	0.33
Red marrow	1.25	0.17	1.84	0.43
Osteogenic cells	1.32	0.25	1.94	0.49
Skin	0.55	0.12	0.77	0.17
Spleen	2.14	0.42	2.76	0.63
Testes	0.69	0.17	0.91	0.21
Thymus	1.06	0.16	1.52	0.32
Thyroid	4.16	2.19	3.66	1.88
Urinary bladder wall	2.80	0.41	2.61	0.33
Uterus	0.97	0.20	1.28	0.26
Total body	0.85	0.15	1.19	0.25
Effective dose equivalent (cGY/37 MBq)	1.65	0.21	2.13	0.36
Effective dose (cGy/37 MBq)	1.36	0.21	1.69	0.26

## Discussion

The imaging findings suggest significant heterogeneity in tumor targeting. While the majority of patients demonstrated evidence of tumor targeting, one patient (no. 8) was negative in spite of tumor size that was adequate for imaging with relatively low IHC and several had relatively low tumor accumulation compared to the normal liver background. This lack of targeting could have been related to antigen heterogeneity and low expression of GPC3. Although our numbers are small, our findings suggest that the degree of tumor uptake is related to GPC3 antigen concentration. It is less likely that internalization caused rapid breakdown of antibody with release from tumor, given that very good localization was seen in several patients. Pre-clinical studies have shown a slow rate of internalization of GPC3 targeted with antibody [17, 18]. Although we believe that internalizing antibodies are likely to be more successful for imaging when labeled with residualizing radionuclides rather than with non-residualizing halogens such as iodine, because of the eventual release of radioiodine after internalization [35, 36], we do note that I-124-labeled antibodies that internalize have been used successfully for tumor imaging [6, 37]. We could not ascribe the lack of visualization in one patient to the damage of the antibody by iodination, since all immunoreactivity assays were acceptable. Furthermore, in seven patients undergoing injection with cold codrituzumab, additional analysis in this study confirmed that the clearance kinetics of the I-124 codrituzumab was similar to that of the parent based on an ELISA assay [27]. For example, no significant differences were observed when we compared the pharmacokinetics and AUC of the cold antibody based on ELISA and the I-124-labeled antibody based on radioactivity measurements; this suggests that the radioiodination did not significantly damage the antibody. Visualization of the thyroid in spite of a thyroid blockade is similar to what has been observed with other iodinated antibodies, while this could be due to poor compliance taking the SSKI, it is more likely that catabolized antibody released free iodine and a small percentage unblocked uptake is sufficient to be visualized given the sensitivity of PET.

The pattern of localization was somewhat atypical for what we have observed in other intact IgG, where peak tumor uptake is not usually seen within the first 24 h and where it is typical to have no localization evident within 4 h of infusion. In this case, we often saw the lesion in our first scanning session within 4 h of injection. This rapid localization may be related to the high vascularity of HCC. The decrease in tumor uptake after peak localization at 24 to 48 h appears to be unusual, although seen in pre-clinical imaging with a Zr-89-labeled antibody targeting the same GPC3 in primary HCC orthotopic xenografts [18].

In the two patients with the highest tumor uptake, we observed the largest drop in tumor concentration following therapy with sorafenib and large amounts of cold codrituzumab that had been administered over several weeks and then again on the day of repeat administration of I-124 codrituzumab. The most prominent decrease was seen in patient 7 (Fig. 5). This drop in concentration in tumor tissues was most likely due to competition at the tumor site and indicated that, as desired, we were “hitting the target,” other possibilities include modulation of antigen or due to a significant tumor response from treatment-induced shrinkage (not supported by CT data).

We were unable to demonstrate that the degree of tumor uptake was related to tumor response, length on study, or length of treatment (data not shown). This may be related to either the lack of efficacy of the combined immunotherapy component or to the small number of patients in our study. Three patients agreed to a third study after progressing on therapy. Interestingly, in those patients, tumor uptake was again present, suggesting that the lack of response was not entirely related to modulation/elimination of antigen-positive cells.

Pharmacokinetics of I-124 codrituzumab was similar to those of other humanized antibodies [5, 13, 38]. The volume of distribution of the central compartment was similar for the various doses and appeared to correspond to plasma volume. There were non-linear pharmacokinetics with larger AUC and slower clearance for injection of I-124 codrituzumab co-infused with cold codrituzumab antibody, although no differences were observed between the 2.5 and 5 mg/kg dose levels. The pharmacokinetic parameters such as Vdss and clearance overlapped with those reported in two prior immunotherapy reports using this same non-radio-labeled antibody [22, 26].

As expected, the injection of the antibody was well tolerated and no serious adverse effects or symptoms could be attributed to the radio-labeled antibody administration. The overall toxicity associated with the injection of immunotherapy dose with sorafenib has been reported separately [27]. Given the pharmacokinetics, biodistribution, and physical properties of I-124, the dosimetry to normal organs was high. Given that we are trying to develop this as a theranostic companion to therapy, the radiation dosimetry is considered acceptable and could probably be decreased further by reducing the injected activity while still maintaining adequate imaging.

## Conclusions

In summary, we have characterized the biodistribution and pharmacokinetics of an I-124 positron-emitting radio-labeled antibody. This study suggests that tumor uptake was related to antigen expression. Furthermore, it shows great heterogeneity of antibody targeting to tumor. While this study showed that it is technically feasible to perform quantitative imaging using I-124 antibody and shows proof of principle that we can successfully image some patients with HCC and determine that we have “hit our target,” we were unable to demonstrate that imaging could be used to identify patients likely to respond in this small subset of patients, in which the combination of codrituzumab and sorafenib did not result in tumor responses. Given the promising tumor imaging results of Zr-89-labeled antibodies, it may be useful to compare Zr-89 codrituzumab with I-124 codrituzumab if future ADC studies are contemplated.

## Additional file

Additional file 1: **Table S1.** H score by Ventana method for GPC3 expression. **Figure S1.** SUVs over normal organs and tissues derived from VOI analysis were divided by SUV in the blood pool at various imaging times. With the exception of the thyroid, which showed increasing accumulation over time and therefore rising tumor-to-blood-pool ratios, activity in other organs had fixed organ-to-blood-pool ratios (spleen and lung) or minimally significantly increased in the liver, kidney, and marrow over time (one-way ANOVA *p* ≤ 0.05, suggesting that accumulation in those organs was accounted for mainly by the blood pool). **Figure S2.** Correlation between SUVmax in tumor and soluble GPC3 values measured by GT30/GT607 pair (A) or GT96/M3C11 pair (B). There is a suggestion of some correlation of uptake to sGPC3 values. **Figure S3.** Pearson correlation between SUVmax in tumor compared to IHC score based on cytoplasm (A) or membrane staining (B). There is a trend to correlation of uptake of antibody expressed in terms of SUV to IHC score. **Figure S4.** No correlation was observed between SUVmax uptake and various clinical outcomes. (DOCX 300 kb)
