# Supplementary material for: I-124 codrituzumab imaging and biodistribution in patients with hepatocellular carcinoma
Source: EJNMMI Res. 2018 Mar 5;8:20. doi: 10.1186/s13550-018-0374-8 (PMC5838028; doi:10.1186/s13550-018-0374-8)
Supplement: Supplementary file 1 — Table S1. H score by Ventana method for GPC3 expression. Figure S1. SUVs over normal organs and tissues derived from VOI analysis were divided by SUV in the blood pool at various imaging times. With the exception of the thyroid, which showed increasing accumulation over time and therefore rising tumor-to-blood-pool ratios, activity in other organs had fixed organ-to-blood-pool ratios (spleen and lung) or minimally significantly increased in the liver, kidney, and marrow over time (one-way ANOVA p ≤ 0.05, suggesting that accumulation in those organs was accounted for mainly by the blood pool). Figure S2. Correlation between SUVmax in tumor and soluble GPC3 values measured by GT30/GT607 pair (A) or GT96/M3C11 pair (B). There is a suggestion of some correlation of uptake to sGPC3 values. Figure S3. Pearson correlation between SUVmax in tumor compared to IHC score based on cytoplasm (A) or membrane staining (B). There is a trend to correlation of uptake of antibody expressed in terms of SUV to IHC score. Figure S4. No correlation was observed between SUVmax uptake and various clinical outcomes. (DOCX 300 kb) [file 13550_2018_374_MOESM1_ESM.docx]

Additional file 1

Table S1. H-score by Ventana method for GPC3 expression

| H score was derived by summing % of cells staining at each intensity (weak, moderate, strong) multiplied by the weighted intensity of staining. | |
| --- | --- |
| Formula of H scores: | |
|  | (1 × [percentage of cells staining at an intensity of 1: weak])  + (2 × [percentage of cells staining at an intensity of 2: moderate])  + (3 × [percentage of cells staining at an intensity of 3: strong]) |

**Figure S1.** SUVs over normal organs and tissues derived from VOI analysis were divided by SUV in blood pool at various imaging times. With the exception of the thyroid, which showed increasing accumulation over time and therefore rising tumor-to-blood-pool ratios, activity in other organs had fixed organ-to-blood-pool ratios (spleen and lung) or minimally significantly increased liver, kidney, and marrow over time (one way ANOVA p<0.05, suggesting that accumulation in those organs was accounted for mainly by blood pool.


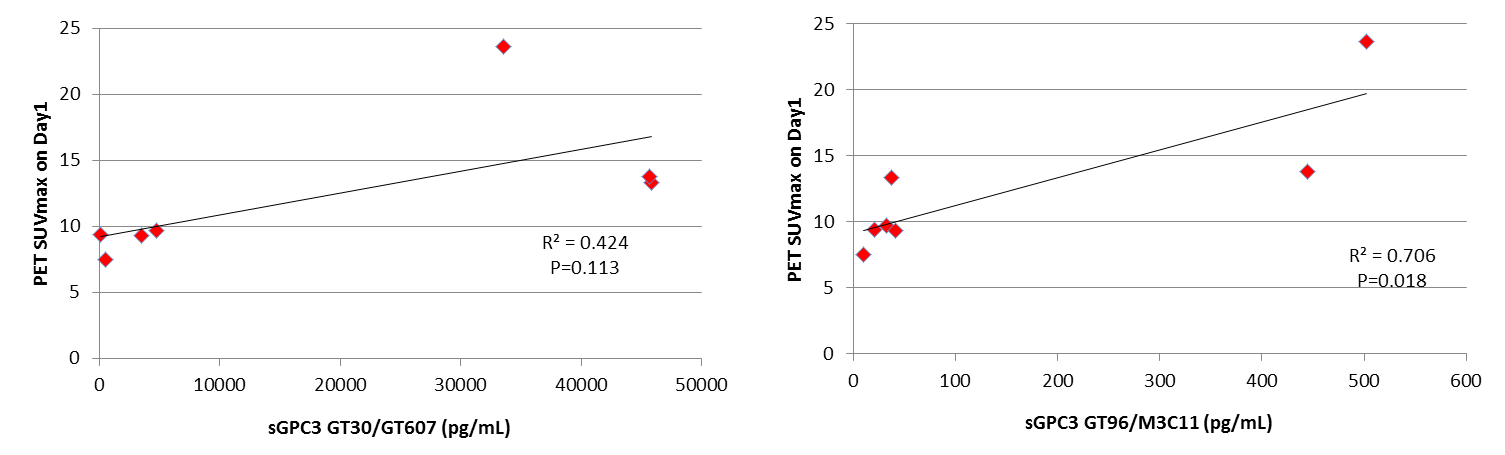

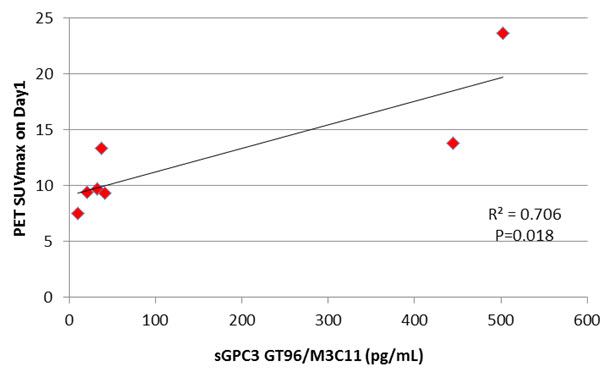


**Figure S2.** Correlation between SUVmax in tumor and soluble GPC3 values measured by GT30/GT607 pair (A) or GT96/M3C11 pair (B). There is a suggestion of some correlation of uptake to sGPC3 values.

**B**

**A**

Figure S3. Pearson correlation between SUVmax in tumor compared to IHC score based on cytoplasm (A) or membrane staining (B). There is a trend to correlation of uptake of antibody expressed in terms of SUV to IHC score.

**Figure S4.** No correlation was observed between SUVmax uptake and various clinical outcomes.
